# *S100A8* gene copy number and protein expression in breast cancer: associations with proliferation, histopathological grade and molecular subtypes

**DOI:** 10.1007/s10549-023-07019-6

**Published:** 2023-07-14

**Authors:** Mathieu Le Boulvais Børkja, Miriam S. Giambelluca, Borgny Ytterhus, Wenche S. Prestvik, Geir Bjørkøy, Anna M Bofin

**Affiliations:** 1 Department of Clinical and Molecular Medicine, Faculty of Medicine and Health Sciences, Norwegian University of Science and Technology, Trondheim, Norway; 2 Department of Clinical Medicine, Faculty of Health Science, UiT- The Arctic University of Norway, Tromsø, Norway; 3 Centre of Molecular Inflammation Research (CEMIR), Faculty of Medicine and Health Sciences, Norwegian University of Science and Technology, Trondheim, Norway; 4 Department of Biomedical Laboraxtory Science, Faculty of Natural Sciences, Norwegian University of Science and Technology, Trondheim, Norway; 5 Science Centre Nordland, Midtre gate 1, Mo i Rana, 8624 Norway

**Keywords:** S100A8, Breast cancer, Copy number, Amplification, MDSC, Protein expression

## Abstract

**Background and aims:**

Amplification of *S100A8* occurs in 10–30% of all breast cancers and has been linked to poorer prognosis. Similarly, the protein S100A8 is overexpressed in a roughly comparable proportion of breast cancers and is also found in infiltrating myeloid-lineage cells, again linked to poorer prognosis. We explore the relationship between these findings.

**Methods:**

We examined *S100A8* copy number (CN) alterations using fluorescence in situ hybridization in 475 primary breast cancers and 117 corresponding lymph nodes. In addition, we studied S100A8 protein expression using immunohistochemistry in 498 primary breast cancers from the same cohort.

**Results:**

We found increased *S100A8* CN (≥ 4) in tumor epithelial cells in 20% of the tumors, increased S100A8 protein expression in 15%, and ≥ 10 infiltrating S100A8 + polymorphonuclear cells in 19%. Both increased *S100A8* CN and protein expression in cancer cells were associated with high Ki67 status, high mitotic count and high histopathological grade. We observed no association between increased *S100A8* CN and S100A8 protein expression, and only a weak association (p = 0.09) between increased CN and number of infiltrating S100A8 + immune cells. Only S100A8 protein expression in cancer cells was associated with significantly worse prognosis.

**Conclusions:**

Amplification of *S100A8* does not appear to be associated with S100A8 protein expression in breast cancer. S100A8 protein expression in tumor epithelial cells identifies a subgroup of predominantly non-luminal tumors with a high mean age at diagnosis and significantly worse prognosis. Finally, S100A8 alone is not a sufficient marker to identify infiltrating immune cells linked to worse prognosis.

## Introduction

Despite significant advances in our understanding of breast cancer (BC) and its treatment, there is still an urgent need for new prognostic and predictive markers to reduce overall mortality due to the disease and morbidity associated with its treatment.

S100A8 and S100A9, in addition to their multitudinous other functions [1], are two of the most abundant alarmins in the human body and can be found in significantly increased quantities across almost all inflammatory conditions [2–4]. Increasingly, they are also recognized for their role in the initiation and progression of cancer [5]. In BC, the gene *S100A8* is found to be amplified in about 10–30% of patients [6–8], and amplification of its chromosomal neighborhood, 1q21.3, has been linked to poorer prognosis in BC patients [8]. Similarly, the protein S100A8 is overexpressed in many common cancers, particularly in BC [9, 10]. Overexpression of S100A8 in cancer cells has been studied in several different populations of BC patients and has consistently been found to be associated with poor clinicopathological features and reduced survival [11–14].

In addition to the pro-tumor effects of S100A8 when expressed in cancer cells which, in BC, so far have mostly been linked to transformation, invasion and migration [15–17], it is thought to contribute further to cancer progression through its association with a loosely defined class of immune-suppressive myeloid cells. These are commonly referred to as Myeloid Derived Suppressor Cells (MDSCs) and can be found in elevated levels in most forms of cancer [18, 19]. S100A8, along with its partner S100A9, has been found to induce the accumulation and differentiation of these cells [20–23]. This has specifically been shown in mouse models of BC, where they also appear to have a role in preparing pre-metastatic niches [21, 24]. Both Drews-Elger et al. and, more recently, Woo et al. have independently reported finding infiltrating S100A8 + immune cells in human BCs, which are associated with reduced survival [13, 25].

While both amplification of *S100A8* and S100A8 expression in BC cells have been studied separately, the relationship between the two remains to be elucidated. Furthermore, the relationship between infiltrating S100A8 + immune cells and increased *S100A8* copy number (CN) or S100A8 expression in tumor cells has not been described in detail. Finally, there is little data on the histopathological features of tumors with increased *S100A8* CN.

To address these points, we used fluorescence in situ hybridization (FISH) to study *S100A8* CN in formalin-fixed, paraffin-embedded tissue (FFPE) from primary BCs and their corresponding axillary lymph node metastases. We also performed immunohistochemistry (IHC), staining for S100A8 in the same primary BCs.

## Materials and methods

### Study population

**Fig. 1:**
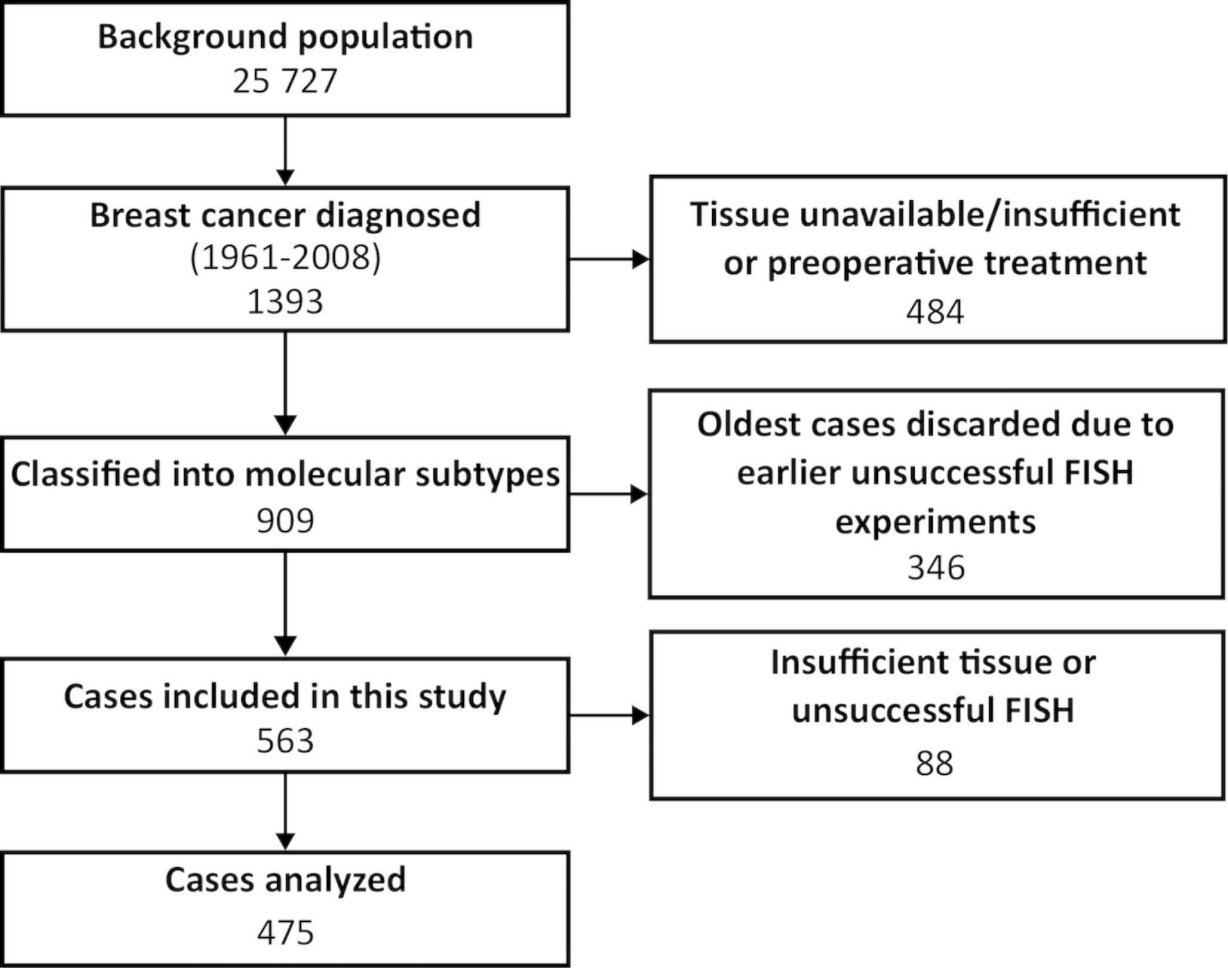
Overview of the study population. Inclusion and exclusion criteria

Between 1956 and 1959, a cohort of 25 727 women from Nord-Trøndelag, Norway, were invited to participate in a population-based program for the early detection of BC [26]. Using information from the Cancer Registry of Norway and the Norwegian Cause of Death Registry, a total of 1396 BC cases were identified in this cohort during the follow-up period from 1961 to 2008 [Fig. 1]. 909 of these were again reclassified into molecular subtypes [27] and followed from time of diagnosis until either death from BC, death from other causes, or the 31st of December 2015.

### Specimen characteristics

Full-face sections from the primary BCs had previously been reclassified into histopathological type and grade in accordance with established guidelines [28, 29]. After that, tissue microarray (TMA) blocks were constructed by extracting three 1 mm tissue cores from the periphery of the FFPE primary BCs and axillary lymph node metastases using a Tissue Arrayer MiniCore with TMA designer2 software (Alphelys). These blocks were then cut into 4 μm thick sections and kept at -20 °C until use. Using IHC and chromogenic in-situ hybridization applied to TMAs in place of gene expression analysis, all cases were then classified into molecular subtypes as previously described (Table 1) [27].

**Table 1:** Classification and distribution of molecular subtypes

	ER + and/or PR+		HER2		Ki67		CK5 + and/or EGFR+	Number included
Luminal A		+	-	< 15%				268/498
Luminal B (HER2^−^)		+	-	≥ 15%				116/498
Luminal B (HER2^+^)		+	+					36/498
HER2 type		-	+					28/498
5NP		-	-			-		12/498
BP		-	-			+		35/498

ER estrogen receptor, PR progesterone receptor, HER2 human epidermal growth factor receptor 2, 5NP 5 negative phenotype, BP basal phenotype, CK5 cytokeratin 5, EGFR epidermal growth factor receptor 1

Only BCs of more recent date, from the 1980s onwards, were included in this study, leaving 563 BCs available for analysis. Of these 563 cases, 169 had axillary lymph node metastases at the time of diagnosis and from which tissue was available.

### Fluorescence in situ hybridization

Following manufacturer instructions, FISH was carried out using a DAKO Histology FISH accessory Kit (Code K 5799). TMA slides were first heated at 60 °C for 1–2 h before dewaxing and rehydrating. Subsequently, they were boiled for 15 min in a pre-treatment solution (Dako) before cooling to room temperature and washing in Dako buffer (2 × 3 min). The slides were then immersed in pepsin solution (37 °C, 30 min) for protein digestion, washed in Dako buffer (2 × 3 min), dehydrated (2 min each, 70%, 85%, and 96% ethanol), and allowed to air-dry. Then, 3 µL of a custom *S100A8* FISH-probe (Empire Genomics) was mixed with 12 µL of hybridization buffer (Empire Genomics) and applied to the slides, which were coverslipped and sealed with coverslip sealant (Dako). They were then placed in a Dako Hybridizer and denatured (83 °C, 3 min) before they were renatured at 37 °C overnight. After this, the slides were rinsed in Stringent Wash Buffer (Dako) (72 °C, 2 min) and Dako wash buffer (2 × 3 min) at room temperature. Next, they were dehydrated (2 min each, 70%, 85%, and 96% ethanol) and dried at 37 °C for 15 min. Finally, 20 µL DAPI (VYSIS Abbott no 06J50-001) was applied to the slides, which were again coverslipped.

### Immunohistochemistry

Immunohistochemistry was performed on 498 of the original 563 primary BCs using a Dako EnVision + Dual Link System-HRP (DAB+) (Cat #4065) following the manufacturer’s protocol. A total of 65 BCs were excluded due to insufficient tissue. TMA slides were first deparaffinized and rehydrated. Epitope retrieval was achieved by boiling the slides for 15 min in citrate buffer pH 6 (Sigma, Cat#C9999) and letting them air-dry before rehydrating and washing them in PBS. The slides were then covered with Dual Endogen Enzyme block (Dako) for 5–10 min at room temperature and washed in PBS (2 × 5 min). After this, anti-human monoclonal mouse IgG_1_ S100A8 antibody (R&D systems, Cat #MAB4570, 1:8000) was applied, and the slides were incubated at 4 °C overnight. The next morning the slides were washed in PBS (3 × 5 min) before the secondary antibody and enzyme were added (Dako Labelled polymer-HRP, 30 min at room temperature) followed by another wash in PBS (3 × 5 min). Subsequently, the slides were covered with a mixture of 20 µL DAB chromogen (Dako) and 1 mL Substrate buffer (Dako) for 5–10 min at room temperature, rinsed with water and contrast stained with hematoxylin. Finally, the slides were dehydrated (2 min each, 70%, 85% and 96% ethanol), cleared using xylene, and coverslips were mounted.

### Scoring and reporting

#### *S100A8* FISH

All samples were analyzed using a Nikon Eclipse 90i fluorescence microscope. The number of fluorescent signals within 20 well-preserved, non-overlapping cancer cell nuclei from a single TMA core was counted for each case. Where multiple tissue cores were available, the one with the best signal quality was chosen. In cases with no noticeable difference in signal quality, the core with the highest apparent count number was selected. Where that again failed to distinguish the cores, one was chosen arbitrarily. Similarly, where cancer cell populations within a single core were heterogeneous with respect to CN, nuclei with the highest CN of *S100A8* were counted. The means *S100A8* CN for each tumor and each metastasis were then calculated. Finally, the cases were divided into two categories according to mean CN: <4 and ≥ 4 CN of *S100A8*. The REMARK criteria for tumor marker studies were followed [30].

#### S100A8 immunohistochemistry

Two different types of S100A8 staining were assessed using a bright-field microscope. Staining within tumor cells was recorded as an estimate of the percentage of positively staining cells among all epithelial tumor cells within a single TMA core. The cases were divided into three categories based on this percentage: < 1%, ≥ 1% < 10%, and ≥ 10%. In addition, within a single TMA core, the absolute number of positively stained infiltrating polymorphonuclear cells was recorded. Cells were regarded as infiltrating if found within the tumor, either in the epithelial or stromal compartment, but not visibly contained in a vessel. This data was used to define two categories: < 10 and ≥ 10 infiltrating S100A8 + polymorphonuclear cells. Again, where more than one TMA core was available, the one with the highest number of staining cells was chosen. The cut-off of 10 cells was chosen arbitrarily.

### Statistical analyses

The differences in tumor characteristics between the defined categories were examined using Pearson’s χ^2^ test. A log-normal accelerated failure time was chosen for the survival analysis because the proportional hazards assumption was inappropriate for some of the included covariates. The log-normal distribution was chosen after fitting the model using several different distributions (log-logistic, log-normal, Weibull, exponential, generalized gamma) and comparing the goodness of fit of each model using both the Akaike and the Bayesian information criteria. In the final model, the impact of the included variables on survival is given as a time ratio with a 95% confidence interval.

## Results

### Fluorescence in situ hybridization

FISH was successful in 475 of the primary tumors and 117 of the axillary lymph node metastases. Eighty-three primary tumors and 52 lymph node metastases were excluded either due to an insufficient amount of tissue available or unsatisfactory FISH.

Table 2 shows patient and tumor characteristics of the 475 cases included in the FISH study, further divided into two categories: those with a mean *S100A8* CN per cancer cell < 4, and those with CN ≥ 4. The mean age at diagnosis in the study population was 76.1 years, and the mean follow-up after diagnosis was 8.7 years. By the end of follow-up 171 (36%) women had died from BC while 255 (54%) had died from other causes.

**Table 2:** Characteristics of the study population according to mean *S100A8* copy number

	Study Population	Mean *S100A8* CN in the primary tumor		
		< 4	≥ 4	p-value (χ2)
N (%)	475	379 (80)	96 (20)	
Mean age at diagnosis, years (SD)	76.1 (8.2)	76.4 (8.3)	75.1 (7.6)	
Mean follow-up after diagnosis, years (SD)	8.7 (6.8)	8.9 (6.8)	8.1 (6.7)	
Deaths from BC (%)	171 (36)	131 (35)	40 (42)	
Deaths from other causes (%)	255 (54)	206 (54)	49 (51)	
*Grade (%)*				
1	48 (10)	42 (11)	6 (6)	< 0.001
2	267 (56)	233 (61)	34 (35)	
3	160 (34)	104 (27)	56 (58)	
*Lymph node metastasis (%)*				
Yes	165 (35)	134 (35)	31 (32)	0.85
No	200 (42)	158 (42)	42 (44)	
Unknown histology	110 (23)	87 (23)	23 (24)	
*Mean S100A8 CN in lymph node metastasis (%)*				
< 4	70 (74)	60 (86)	14 (58)	0.005
≥ 4	24 (26)	10 (14)	10 (42)	
*Molecular subtype (%)*				
Luminal A	251 (53)	212 (56)	39 (41)	0.002
Luminal B (HER2-)	114 (24)	92 (24)	22 (23)	
Luminal B (HER2+)	37 (8)	26 (7)	11 (11)	
HER2 type	25 (5)	17 (5)	8 (8)	
5NP	11 (2)	10 (3)	1 (1)	
BP	36 (8)	21 (6)	15 (16)	
*Histologic subtype (%)*				
Ductal (NOS)	324 (68)	258 (68)	66 (69)	0.3
Lobular	64 (13)	55 (15)	9 (9)	
Other	87 (18)	66 (17)	21 (22)	
*Ki67 high / low (%)*				
Ki67 < 15%	286 (60)	239 (63)	47 (49)	0.03
Ki67 ≥ 15%	188 (40)	139 (37)	49 (51)	
Mitoses / 10 HPF, median (IQR p25, p75)	5 (1, 12)	4 (1, 11)	9 (3, 17)	
*Mitoses / 10 HPF, quartiles (%)*				
≤ 1	114 (27)	100 (29)	14 (18)	0.003
*>* 1, ≤ 5	110 (26)	97 (28)	13 (16)	
*>* 5, ≤ 12	102 (24)	79 (23)	23 (29)	
*>* 12	102 (24)	73 (21)	29 (37)	

N number of patients, SD standard deviation, BC breast cancer, HER2 human epidermal growth factor receptor 2, 5NP 5 negative phenotype, BP basal phenotype, HPF high-power field, IQR interquartile range

**Fig. 2:**
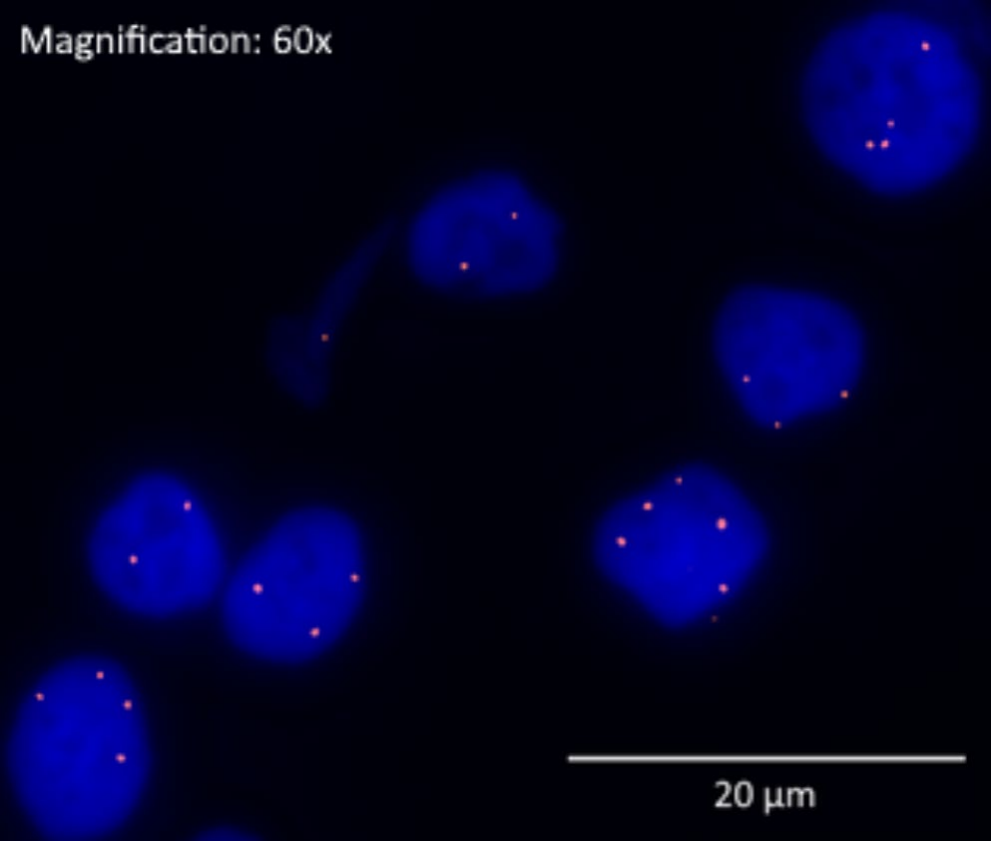
Fluorescence in situ hybridization for *S100A8* at 60x magnification, scale bars: 20 μm, showing increased copy number of *S100A8* (red dots) in tumor cell nuclei stained with Dapi (blue)

*S100A8* was found to be amplified exclusively within cancer cells [Fig. 2], where it was found in a pattern consistent with extrachromosomal amplification.

### *S100A8* CN and molecular subtypes

Increased *S100A8* CN was found across all molecular subtypes. Among luminal tumors increased *S100A8* CN was found in 39/251 (16%) Luminal A, 22/114 (18%) Luminal B, and 11/37 (30%) Luminal B (HER2^+^) tumors. Among the non-luminal tumors, it was found in 8/25 (32%) HER2 tumors, 1/11 (9%) 5NP, and 15/36 (42%) BP tumors (p = 0.002).

### *S100A8* CN, proliferation and histopathological grade

Tumors with increased *S100A8* CN tended to be of higher histopathological grade. Of these tumors 6/96 (6%) were grade 1, 34/96 (35%) were grade 2, and 56/96 (58%) were grade 3 (p < 0.001). *S100A8* CN increase was also significantly associated with increased proliferation. Forty-nine of 96 (51%) of the tumors with *S100A8* CN ≥ 4 were Ki67 high (p = 0.03).

### *S100A8* CN in primary tumors and lymph node metastases

Of the 475 cases with successful FISH, 165 (35%) had confirmed metastases to axillary lymph-nodes at the time of diagnosis. *S100A8* CN ≥ 4 was found in 96/475 (20%) cases. Copy number data for cases with lymph node metastases was available for 94 cases and 24/94 (25%) showed *S100A8* CN ≥ 4 in the primary tumor. Of these, 10/24 (42%) also had increased *S100A8* CN in the lymph-node metastasis while 14/24 (58%) did not (p = 0.005).

### Immunohistochemistry

Table 3 shows patient and tumor characteristics of the 498 cases from the same study population, with successful IHC-staining for S100A8, divided into three categories (< 1%, ≥ 1% < 10%, ≥ 10%) based on the proportion of epithelial tumor cells staining positively for the protein S100A8. The mean age at diagnosis was 75.9 years while the mean follow-up time was 9.2 years. By end of follow-up 167/498 (34%) of the women in the study population had died of BC while 279/498 (56%) had died from other causes.

**Table 3:** Characteristics of the study population according to the proportion of S100A8 staining tumor epithelial cells

	Study Population			Cytoplasmic S100A8 staining		
		< 1%	≥ 1%, < 10%		≥ 10%	p-value (χ2)
N (%)	498	420 (84)		47 (9)	31 (6)	
Mean age at diagnosis, years (SD)	75.9 (8.5)	75.5 (8.4)		76.3 (8.4)	79.9 (9.5)	
Mean follow-up after diagnosis, years (SD)	9.2 (7.1)	9.6 (7.1)		8.3 (6.7)	4.7 (4.9)	
Deaths from BC (%)	167 (34)	134 (32)		14 (30)	19 (61)	
Deaths from other causes (%)	279 (56)	241 (58)		26 (55)	12 (39)	
*Grade (%)*						
1	54 (11)	52 (12)		2 (4)	0 (0)	< 0.001
2	287 (58)	261 (62)		19 (40)	7 (23)	
3	155 (31)	105 (25)		26 (55)	24 (77)	
*Lymph node metastasis (%)*						
Yes	169 (34)	134 (32)		22 (47)	9 (29)	0.08
No	226 (45)	198 (47)		19 (40)	13 (42)	
Unknown histology	103 (21)	88 (21)		6 (13)	9 (29)	
*Molecular subtype (%)*						
Luminal A	268 (54)	247 (59)		17 (36)	4 (13)	< 0.001
Luminal B (HER2-)	116 (23)	106 (25)		8 (17)	2 (7)	
Luminal B (HER2+)	36 (7)	30 (7)		5 (11)	1 (3)	
HER2 type	28 (6)	12 (3)		8 (17)	8 (27)	
5NP	12 (2)	4 (1)		5 (11)	3 (10)	
BP	35 (7)	19 (5)		4 (9)	12 (40)	
*Histologic subtype (%)*						
Ductal (NOS)	351 (70)	294 (70)		35 (75)	22 (71)	0.4
Lobular	65 (13)	60 (14)		3 (6)	2 (6)	
Other	82 (16)	66 (16)		9 (19)	7 (23)	
*Ki67 high / low (%)*						
Ki67 < 15%	301 (60)	268 (64)		25 (53)	8 (26)	< 0.001
Ki67 ≥ 15%	194 (39)	150 (36)		22 (47)	22 (71)	
Mitoses / 10 HPF, median (IQR p25, p75)	5 (1, 12)	4 (1, 9)		9 (2, 16)	14 (8, 21)	
*Mitoses / 10 HPF, quartiles (%)*						
≤ 1	136 (27)	127 (30)		7 (15)	2 (6)	< 0.001
*>* 1, ≤ 5	134 (27)	121 (29)		9 (19)	4 (13)	
*>* 5, ≤ 12	116 (23)	94 (22)		13 (28)	9 (29)	
*>* 12	112 (22)	78 (19)		18 (38)	15 (48)	
*Number of infiltrating* S100A8 *+ PMN cells (%)*						
< 10	405 (81)	342 (81)		38 (81)	25 (81)	0.99
≥ 10	93 (19)	78 (19)		9 (19)	6 (19)	

N number of patients, SD standard deviation, BC breast cancer, HER2 human epidermal growth factor receptor 2, 5NP 5 negative phenotype, BP basal phenotype, HPF high-power field, IQR interquartile range

**Fig. 3:**
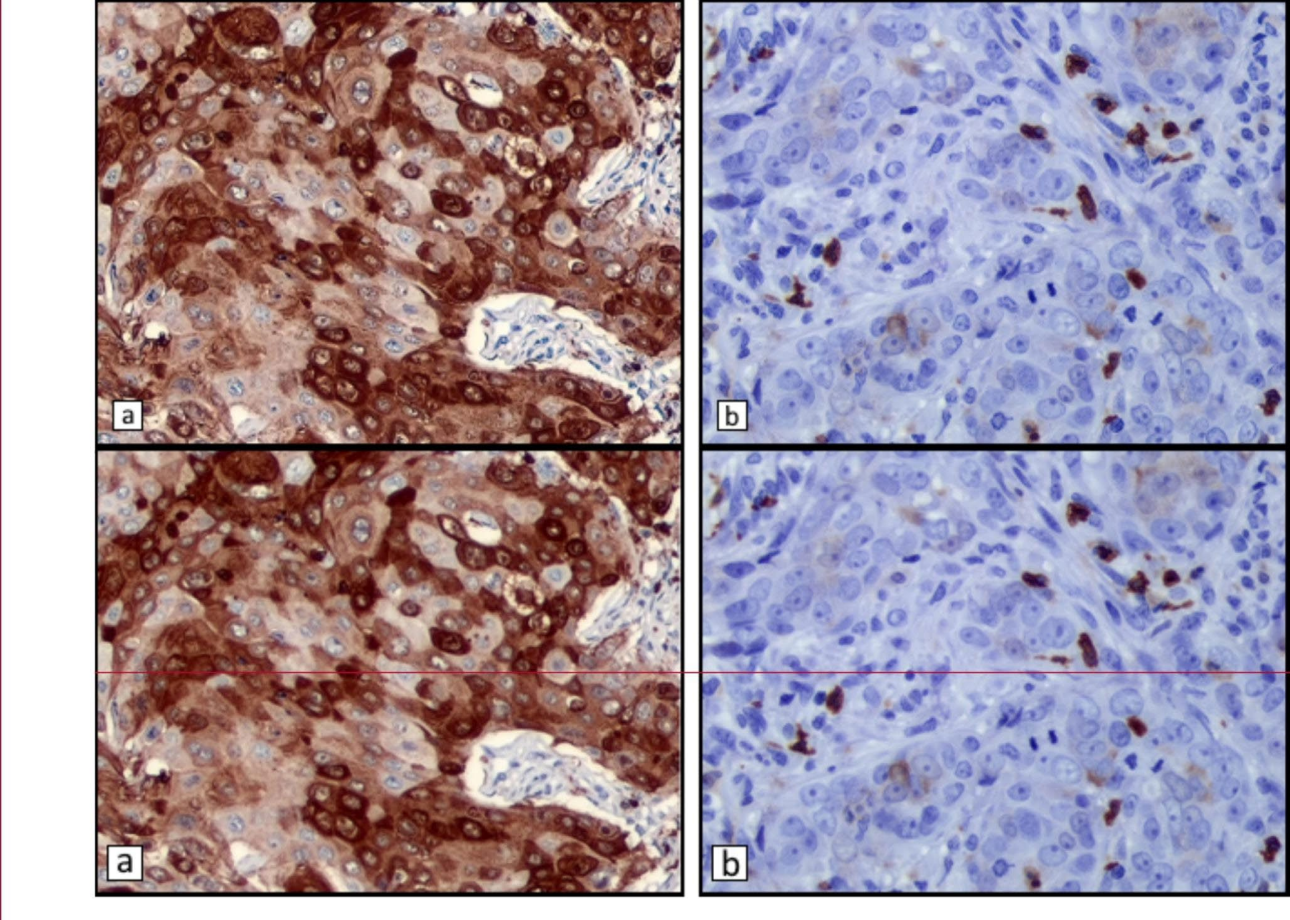
Immunohistochemical staining showing positive cytoplasmic staining at 400x magnification in (**a**) cancer cells, and (**b**) infiltrating polymorphonuclear cells

Positive S100A8 staining was observed both in epithelial tumor cells and in polymorphonuclear (PMN) cells. In both cell types, staining was diffusely cytoplasmic. [Figure 3] shows representative images of this type of staining. Patient and tumor characteristics according to the proportion of S100A8 + tumor epithelial cells are presented in Table 3 and explained in this section. Results for S100A8 in PMNs are presented in Table 4.

**Table 4:** Characteristics of the study population according to the presence of ≥ 10 infiltrating S100A8 + PMN cells

	Study Population	Infiltration of S100A8 + cells		
		< 10	≥ 10	p-value (χ2)
N (%)	498	405 (81)	93 (19)	
Mean age at diagnosis, years (SD)	75.9 (8.5)	76.0 (8.5)	75.1 (8.5)	
Mean follow-up after diagnosis, years (SD)	9.2 (7.1)	9.1 (7.1)	9.6 (7.1)	
Deaths from BC (%)	167 (34)	133 (33)	34 (37)	
Deaths from other causes (%)	279 (56)	233 (58)	46 (49)	
*Grade (%)*				
1	54 (11)	49 (12)	5 (5)	< 0.001
2	287 (58)	247 (61)	40 (43)	
3	155 (31)	107 (27)	48 (52)	
*Lymph node metastasis (%)*				
Yes	169 (34)	134 (33)	35 (38)	0.7
No	226 (45)	186 (46)	40 (43)	
Unknown histology	103 (21)	85 (21)	18 (19)	
*Molecular subtype (%)*				
Luminal A	268 (54)	234 (58)	34 (37)	< 0.001
Luminal B (HER2-)	116 (23)	94 (23)	22 (24)	
Luminal B (HER2+)	36 (7)	25 (6)	11 (12)	
HER2 type	28 (7)	18 (5)	10 (11)	
5NP	12 (2)	9 (2)	3 (3)	
BP	35 (7)	22 (5)	13 (14)	
*Histologic subtype (%)*				
Ductal (NOS)	351 (70)	289 (71)	62 (67)	0.04
Lobular	65 (13)	57 (14)	8 (9)	
Other	82 (16)	59 (15)	23 (25)	
*Ki67 high / low (%)*				
Ki67 < 15%	301 (60)	264 (65)	37 (40)	< 0.001
Ki67 ≥ 15%	194 (39)	138 (34)	56 (60)	
Mitoses / 10 HPF, median (IQR p25, p75)	5 (1, 12)	4 (1, 9)	9 (4, 20)	
*Mitoses / 10 HPF, quartiles (%)*				
≤ 1	136 (27)	123 (30)	13 (14)	< 0.001
*>* 1, ≤ 5	134 (27)	116 (29)	18 (19)	
*>* 5, ≤ 12	116 (23)	89 (22)	27 (29)	
*>* 12	111 (22)	76 (19)	35 (38)	

N number of patients, SD standard deviation, BC breast cancer, HER2 human epidermal growth factor receptor 2, 5NP 5 negative phenotype, BP basal phenotype, HPF high-power field, IQR interquartile range

### S100A8 expression in primary tumors

S100A8 + staining in ≥ 1% of tumor cells was observed in 78/498 (16%) of cases. Of these, 47/498 (9%) had between ≥ 1% <10% positive-staining tumor epithelial cells, while 31/498 (6%) had ≥ 10%. The mean age at diagnosis among the latter group of patients was 79.9 years, 4 years higher than that of the study population as a whole and 19/31 (61%), had died from BC by the end of follow-up.

### S100A8 and molecular subtypes

Tumors with S100A8 + tumor epithelial cells were found among all molecular subtypes. There was a gradual shift away from luminal types in favor of non-luminal types with increasing levels of S100A8 staining. Among tumors with ≥ 10% S100A8 + tumor epithelial cells, we found 4/268 (1%) Luminal A, 2/116 (2%) Luminal B, and 1/36 (3%) Luminal B (HER2^+^) tumors, whereas we found 8/28 (29%) HER2, 3/12 (25%) 5NP, and 12/35 (34%) BP tumors expressed S100A8 in ≥ 10% of tumor epithelial cells. Notably, of all tumors with S100A8 + ≥ 10%, 23/31 (74%) were of non-luminal types.

### S100A8, proliferation and histopathological grade

Higher proportions of S100A8 + tumor epithelial cells were significantly associated both with increased proliferation and with higher histopathological grade. Of tumors with ≥ 10% S100A8 + cancer cells 22/31 (71%) were Ki67 high. In the same group there were no grade 1 tumors, 7/31 (23%) grade 2 tumors, while the remaining 24/31 (77%) were grade 3.

### S100A8 and lymph node metastases

A weak association between S100A8 staining in tumor epithelial cells and axillary lymph-node status at the time of diagnosis was observed (p = 0.08). Metastases to axillary lymph-nodes were more frequent in the intermediate group (≥ 1% <10%), 22/47 (47%). Only 9/31 (29%) of tumors with ≥ 10% positive staining cells had metastases to axillary lymph-nodes at time of diagnosis.

### S100A8 in cancer cells and in infiltrating PMN cells

No association was observed between the proportion S100A8 + tumor epithelial cells and the number of infiltrating S100A8 + PMN cells.

### S100A8 in infiltrating PMN cells

Ten or more infiltrating S100A8 + PMN cells were found in 93/498 (19%) cases. These tumors were found among all molecular subtypes: 67/93 (72%) among luminal types and 26/93 (28%) among non-luminal types. They tended to be of high histopathological grade with 48/93 (52%) tumors being grade 3 and 40/93 (40%) being grade 2. No significant associations between the presence of these cells and metastases to axillary lymph-nodes were observed (Table 4).

### *S100A8* CN and protein expression

As shown in Table 5, no significant association was observed between mean *S100A8* CN/tumor epithelial cell and the proportion of S100A8 + cells. There was, however, a weak association between mean *S100A8* CN and the number of infiltrating S100A8 + PMN cells (p = 0.09). A somewhat higher proportion of tumors with increased *S100A8* CN also had ≥ 10 S100A8 + PMN cells.

**Table 5:** Relationship between mean *S100A8* copy number (CN) and protein expression

			Mean *S100A8* CN		
			< 4	≥ 4	p-value (χ2)
*Proportion of* S100A8 *+ cancer cells (%)*					
< 1%	356 (83)		292 (84)	64 (81)	0.5
≥ 1% < 10%	42 (10)		35 (10)	7 (9)	
≥ 10%	30 (7)		22 (6)	8 (10)	
*Number of infiltrating* S100A8 *+ PMN cells (%)*					
< 10	348 (81)		289 (83)	60 (75)	0.09
≥ 10	80 (19)		59 (17)	20 (25)	

### Survival analysis

**Fig. 4:**
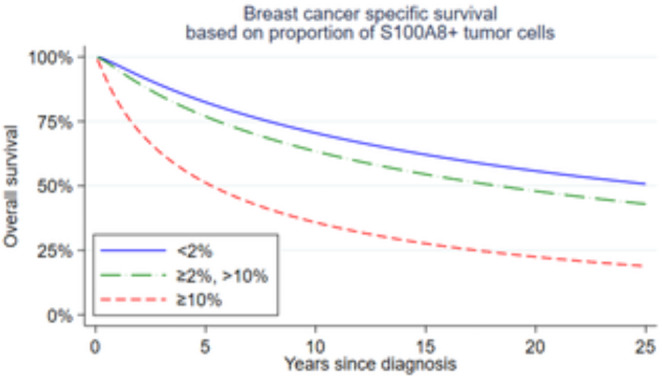
Breast cancer specific survival based on proportion of S100A8 + tumor epithelial cells using a log-normal AFT model. Time ratios as well as p-values are given in Table 6

Figure 4. Relative prognosis was estimated by using a log-normal accelerated failure time model. The results of such an analysis are shown in Table 6 for four different variables, each previously described. A plot for one of these variables is shown in Fig. 4. No significant differences in survival were observed between cases with and without increased *S100A8* CN, neither in the primary tumor nor in axillary lymph-node metastases. Neither was the presence of ≥ 10 S100A8 + PMN cells in the tumor associated with significantly different patient prognosis. However, patients with tumors with ≥ 10% S100A8 + tumor epithelial cells did have significantly poorer survival (p < 0.001). The time ratio for this variable was estimated to be 0.2 (95% CI 0.10–0.42), meaning that the average estimated lifespan of patients in this group, from time of diagnosis, was 20% of that of cases with less than 1% of S100A8 + tumor epithelial cells. As is shown in Table 7 this time ratio remained significantly different from 1 when adjusted for age, grade, histologic subtype, molecular subtype and Ki67 status.

**Table 6:** Parameter estimates and 95% confidence intervals for log-normal accelerated failure time model

Variable	Time ratio (95% CI)	P
*Mean S100A8 CN (primary tumor)*		
< 4	Reference	
≥ 4	0.77 (0.47–1.24)	0.28
*Mean S100A8 CN (lymph node metastases)*		
< 4	Reference	
≥ 4	0.60 (0.30–1.20)	0.15
*Proportion of* S100A8 + *tumor epithelial cells*		
< 1%	Reference	
≥ 1%, < 10%	0.71 (0.37–1.36)	0.30
≥ 10%	0.20 (0.10–0.42)	< 0.001
*Number of infiltrating* S100A8 + *PMN cells*		
< 10	Reference	
≥ 10	0.83 (0.51–1.37)	0.46

TR time ratio, CI confidence interval, CN copy number, PMN polymorphonuclear

**Table 7:** Parameter estimates for different log-normal AFT models with proportion of S100A8 + tumor epithelial cells as the main covariate, unadjusted and adjusted for age, grade, histologic subtype, molecular subtype and Ki67 status

	Proportion of S100A8 + tumor cells		
	< 1%	≥ 1%, < 10%	≥ 10%
TR unadjusted (95% CI)	Reference	0.71 (0.37–1.36)	0.20 (0.10–0.42)
TR adjusted for age (95% CI)	Reference	0.78 (0.43–1.39)	0.23 (0.12–0.43)
TR adjusted for grade (95% CI)	Reference	0.91 (0.47–1.77)	0.30 (0.14–0.61)
TR adjusted for histological subtype (95% CI)	Reference	0.66 (0.34–1.27)	0.20 (0.10–0.41)
TR adjusted for molecular subtype (95% CI)	Reference	1.05 (0.54–2.05)	0.39 (0.18–0.86)
TR adjusted for Ki67 status (95% CI)	Reference	0.78 (0.41–1.49)	0.25 (0.12–0.51)

TR time ratio, CI confidence interval

## Discussion

The aim of this study was to examine the relationship between *S100A8* CN increase and S100A8 protein expression in BC epithelial cells and infiltrating PMN cells, as well as to explore associations between each of these with proliferation, histopathological grade, molecular subtypes and prognosis. We found that both S100A8 protein expression in tumor cells and, to a lesser extent, *S100A8* CN increase, were associated with high tumor grade and increased proliferation. S100A8 protein expression was strongly associated with non-luminal tumors, and in particular with HER2-type and BP tumors. While S100A8 protein expression in at least 10% of epithelial tumor cells was associated with poor survival, CN increase of *S100A8* was not. We observed no association between *S100A8* CN increase and S100A8 protein expression in tumor cells and only a weak association (p = 0.09) between increased *S100A8* CN and the presence of 10 or more S100A8 + infiltrating PMN cells.

The incidence of *S100A8* CN increase we report here is congruent with the incidence of *S100A8* amplification found in other data sets. Using cBioPortal [31, 32] to explore the Molecular Taxonomy of Breast Cancer International Consortium (METABRIC) data set reveals that *S100A8* is amplified in 21% of the tumors included therein [6, 7]. Similarly, Goh et al. found *S100A8* amplification in between 10 and 30% of tumors in the TCGA breast cancer data set depending on molecular subtype [8].

In the same paper Goh et al. also found that amplification of a profile of 17 genes on chromosome 1q21.3, *S100A8* included, was associated with worse survival and proposed a mechanism involving a reciprocal feedback loop between S100A7, S100A8, S100A9 and IRAK1. A possible reason we did not observe such an association in our population might be that the presence of this amplification alters sensitivity to adjuvant therapy, which the women included in our study for the most part would not have received. Of note, Lang et al. has reported that targeted silencing of the *S100A8* gene by miR-24 increases chemotherapy sensitivity of endometrial carcinoma cells to paclitaxel [33], while Li et al. found that the level of S100A8 expression was superior to molecular subtyping in predicting chemo responses in 120 cases of BC patients [34].

It should also be noted that the amplifications involved in these studies encompass more genes than simply *S100A8*. The FISH probe used in the present study, for example, is ~ 168Kb long and covers six genes (*PGLYRP3*, *PGLYRP4*, *S100A9*, *S100A12*, *S100A7A*) in addition to *S100A8*, making it difficult to attribute specific findings directly to any one gene.

Our findings regarding S100A8 protein expression in BC cells are well in concordance with previous studies [11–13]. Like our findings, Woo et al. reported on S100A8 staining both in BC cells as well as in infiltrating immune cells. However, unlike us, they found that both types of staining were linked to poorer prognosis. In a different study on the same population, we examined another potential marker for MDSCs, arginase-1 (ARG1), and found that 27/92 (29%) of the tumors with significant infiltration of S100A8 + PMNs also had significant infiltration of ARG1 + PMNs. The latter, however, were significantly associated with poorer prognosis (unpublished data). We therefore propose that S100A8 alone may not be the most appropriate surrogate marker for MDSCs due to its abundant expression in different neutrophil and monocyte subpopulations.

Interestingly, all these studies found S100A8 staining exclusively in the cytoplasm of tumor cells. This may be a limitation of the methods applied given that S100A8 has both known extracellular and intranuclear functions associated with cancer [5, 16]. Furthermore, the function and stability of S100A8 is highly dependent on S100A9, and while the genes are generally coamplified, the proteins are not always coexpressed [12]. In future studies it would therefore be an advantage to look at the expression of both proteins.

We observed that women in the group with the largest proportion of S100A8 + tumor epithelial cells had a mean age at diagnosis of 79.9 years, 4 years higher than the mean age at diagnosis of the study population, and that this group had a worse prognosis even after adjusting for age. This is remarkable given that 74% of these tumors were of non-luminal types which are relatively more common in younger women. S100A8 expression may be a response to increasing levels of reactive oxygen and nitrogen species in these tumors, of which both S100A8 and S100A9 are excellent scavengers [35]. In addition, S100A8 and S100A9 are age associated damage-associated molecular patterns (DAMPs) and they, along with MDSCs, have been connected to chronic inflammation during aging [36, 37].

The 563 tumors included in this study came from a well-described, and relatively homogeneous cohort of Norwegian breast cancer patients who were in most cases followed until time of death [26, 27]. Data on patient outcome was taken from high quality national registries [38, 39]. Due to either advanced age at diagnosis, or the time period in which they were diagnosed, most of these women would not have been offered the adjuvant therapy, which is common today, allowing us to study a near-natural course of the disease after surgery. The tumors were collected over a period of more than 40 years and were thus subject to varying pre-analytical conditions. This makes it difficult to study mRNA expression in these BCs. The findings should also be verified in a larger number of non-luminal BCs.

In conclusion, though both the amplification of *S100A8* and the expression of the protein in BC cells may identify BCs with more malignant characteristics, they appear to be unrelated phenomena.

## Data Availability

Data are available upon reasonable request. The datasets included in the current study are not publicly available but are available from the corresponding author on reasonable request.
